# The complete mitochondrial genome sequence of *Sinocyclocheilus jii* (Cypriniformes: Cyprinidae) and phylogenetic implications

**DOI:** 10.1080/23802359.2017.1375878

**Published:** 2017-09-09

**Authors:** Chunqing Li, Xiaohan Xiang, Tiao Ning, Shanyuan Chen, Heng Xiao

**Affiliations:** a School of Life Sciences, Yunnan University, Kunming, China;; b Xiangxi Vocational and Technical College for Nationalities, Jishou, China;; c College of Agricultural Sciences, Kunming University, Kunming, China;; d Key Laboratory for Animal Genetic Diversity and Evolution of High Education in Yunnan Province, Yunnan University, Kunming, China

**Keywords:** Mitochondrial genome sequence, *Sinocyclocheilus jii*, Cyprinidae

## Abstract

In this study, we first determined the complete mitochondrial genome sequence of *Sinocyclocheilus jii*, which is an endemic species to Southwestern China. The complete mitochondrial genome is 16,577 bp in length, consisting of 37 genes coding for 13 proteins, two rRNAs, 22 tRNAs, and one control region. Its gene arrangement pattern was identical to that of most vertebrates. Phylogenetic analysis using mitochondrial genomes of 11 species showed that nine *Sinocyclocheilus* species clustered as one monophyletic clade and *S. jii* was the most basal species on the phylogenetic tree of the *Sinocyclocheilus* fishes.

*Sinocyclocheilus jii* belonging to the genus *Sinocyclucheilus* (Cypriniformes, Cyprinidae) is a freshwater fish that distributes only in the karst landform areas within Fuchuan County and Gongcheng County, Guangxi, China (Zhang and Dai 1992; Zhao and Zhang 2009). In this investigation, we would first report the complete mitochondrial genome (mitogenome) sequence of *S. jii*, which will provide useful molecular data for conservation biology, genetics and evolutionary studies on this species and its closely related species.

The specimen of *S. jii* under study was collected in Gongcheng, Guangxi, China (25.2°N, 110.9°E). The whole specimen was preserved in 95% alcohol and registered in the Zoological Specimen Museum of Yunnan University under the voucher number YNUSJ201308060038. All the experiments, including genomic DNA extraction, applied primers, PCR and sequencing, were carried out by following the standard laboratory procedures described by Wu et al. (2010). The protein-coding genes and ribosomal RNA genes were identified using DOGMA (Wyman et al. 2004), through comparing their similarity to previously reported mitochondrial genomes of *Sinocyclocheilus grahami* (Wu et al. 2010). The 22 tRNA genes of *S. jii* can fold into a typical cloverleaf secondary structure that was estimated by the program tRNAscan-SE (Lowe and Eddy 1997).

The complete mitogenome sequence of *S. jii* had been deposited in the GenBank with an accession number MF100765. The mitogenome was 16,577 bp in size, including 13 protein-coding (PCGs), two ribosomal RNA (rRNA), 22 transfer RNA (tRNA) genes, and one control region (D-loop). All mitochondrial genes of *S. jii* showed the typical gene arrangement conforming to the vertebrate consensus (Boore 1999). All genes were encoded on the H-strand, except for the ND6 gene and eight tRNA genes (tRNA-Gln, tRNA-Ala, tRNA-Asn, tRNA-Cys, tRNA-Tyr, tRNA-Ser (UCN), tRNA-Glu, and tRNA-Pro) which were encoded on the L-strand. Twelve of the PCGs began with ATG, but COI gene started with GTG. Most PCGs used complete (TAA or TAG) stop codon, while COII, ND4, and Cyt b genes ended with incomplete (T-) stop codon. The overall base composition in descending order for *S. jii* is A (31.77%), C (27.00%), T (25.00%), and G (16.23%) with 43.23% GC content.

In order to acquire some implication about phylogenetic position of *S. jii* within the *Sinocyclocheilus* species, mitogenome sequence of *S. jii* in this study, together with previously reported mitogenome sequences of eight *Sinocyclocheilus* fishes and two outgroup species from GenBank were used to perform phylogenetic analysis. Phylogenetic tree of 11 Cyprinidae fishes was built with MrBayes (Ronquist and Huelsenbeck 2003) and RAxML (Stamatakis 2006). Both the two methods generated the identical phylogenetic tree topologies (Figure 1). In the results, nine *Sinocyclocheilus* species clustered as one monophyletic clade with strong supports. The phylogenetic results strongly supported that *S. jii* was the most basal species among the *Sinocyclocheilus* species.

**Figure 1.**
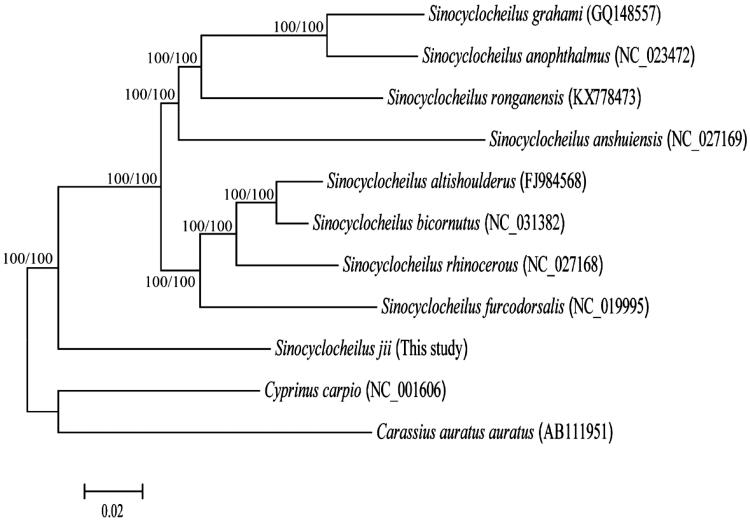
Phylogenetic relationships of 11 Cyprinidae fishes by Bayesian and maximum-likelihood (ML) methods based on complete mitochondrial genome sequences. The accession numbers for each species are indicated after the scientific names. Numbers in the nodes represent the posterior probability for Bayesian analysis and bootstrap value for ML analysis.
